# Metabolic-associated fatty liver disease: a selective review of pathogenesis, diagnostic approaches, and therapeutic strategies

**DOI:** 10.3389/fmed.2024.1291501

**Published:** 2024-01-23

**Authors:** Mohammad Habibullah, Khaleed Jemmieh, Amr Ouda, Mohammad Zulqurnain Haider, Mohammed Imad Malki, Abdel-Naser Elzouki

**Affiliations:** ^1^College of Medicine, QU Health, Qatar University, Doha, Qatar; ^2^Internal Medicine Department, Hamad General Hospital, Doha, Qatar; ^3^Weill Cornell Medical Qatar, Doha, Qatar

**Keywords:** metabolic associated fatty liver disease (MAFLD), non-alcoholic fatty liver disease (NAFLD), diagnostic criteria, hepatic steatosis, nonalcoholic steatohepatitis (NASH), epidemiology, clinical manifestations, pathogenesis

## Abstract

**Background:**

Metabolic associated fatty liver disease (MAFLD) is a novel terminology introduced in 2020 to provide a more accurate description of fatty liver disease associated with metabolic dysfunction. It replaces the outdated term nonalcoholic fatty liver disease (NAFLD) and aims to improve diagnostic criteria and tailored treatment strategies for the disease. NAFLD, the most prevalent liver disease in western industrialized nations, has been steadily increasing in prevalence and is associated with serious complications such as cirrhosis and hepatocellular carcinoma. It is also linked to insulin resistance syndrome and cardiovascular diseases. However, current studies on NAFLD have limitations in meeting necessary histological endpoints.

**Objective:**

This literature review aims to consolidate recent knowledge and discoveries concerning MAFLD, integrating the diverse aspects of the disease. Specifically, it focuses on analyzing the diagnostic criteria for MAFLD, differentiating it from NAFLD and alcoholic fatty liver disease (AFLD), and exploring the epidemiology, clinical manifestations, pathogenesis, and management approaches associated with MAFLD. The review also explores the associations between MAFLD and other conditions. It discusses the heightened mortality risk associated with MAFLD and its link to chronic kidney disease (CKD), showing that MAFLD exhibits enhanced diagnostic accuracy for identifying patients with CKD compared to NAFLD. The association between MAFLD and incident/prevalent CKD is supported by cohort studies and meta-analyses.

**Conclusion:**

This literature review highlights the importance of MAFLD as a distinct terminology for fatty liver disease associated with metabolic dysfunction. The review provides insights into the diagnostic criteria, associations with CKD, and management approaches for MAFLD. Further research is needed to develop more accurate diagnostic tools for advanced fibrosis in MAFLD and to explore the underlying mechanisms linking MAFLD with other conditions. This review serves as a valuable resource for researchers and healthcare professionals seeking a comprehensive understanding of MAFLD.

## Background

Since 2020, a novel terminology known as metabolic associated fatty liver disease (MAFLD) has been developed, introducing its own set of criteria (1). This new acronym aims to provide a more precise description of fatty liver disease associated with metabolic dysfunction, replacing the outdated term nonalcoholic fatty liver disease (NAFLD). The development of improved diagnostic criteria is crucial for better understanding and stratification of the disease, enabling the implementation of tailored treatment strategies targeting specific pathways affected by MAFLD.

The proposal to replace the term “non-alcoholic fatty liver disease (NAFLD)” with “metabolic (dysfunction)-associated fatty liver disease (MAFLD)” marks a significant advancement in the field. This change, suggested by an international panel, has garnered widespread support, with numerous studies across the United States, Europe, and Asia providing substantial evidence of the superiority of MAFLD criteria over NAFLD criteria in various aspects of fatty liver diseases (2).

The existing nomenclature, with its emphasis on the absence of alcohol, inadvertently stigmatizes individuals with fatty liver disease by insinuating a connection to alcohol use, even when such a connection is absent. This stigma can lead to misconceptions and discrimination, affecting patients’ well-being and hindering effective communication between healthcare providers and individuals with the condition (3).

NAFLD encompasses hepatic steatosis when all other causes, such as excessive alcohol consumption, have been ruled out. The condition is further classified into nonalcoholic fatty liver (NAFL) or nonalcoholic steatohepatitis (NASH), depending on the presence of liver inflammation, which is observed only in NASH (4, 5). The original definition was established in 1980 and has remained unaltered since then. However, the prevalence of NAFLD has been steadily increasing, making it the most prevalent liver disease in western industrialized nations, affecting approximately 1 billion individuals worldwide (6, 7). The majority of NAFLD cases are diagnosed in individuals in their 40s or 50s (8), with no apparent gender bias. Furthermore, NAFLD appears to be more prevalent among Hispanic Americans compared to White or Black Americans (9). This is of particular concern since NASH can progress to cirrhosis, hepatocellular carcinoma (HCC), and ultimately result in mortality, placing a significant burden on individuals, families, societies, and healthcare systems (10). In addition, NAFLD has been found to be associated with insulin resistance syndrome (presently termed metabolic syndrome) (11). It is well established that metabolic syndrome is a risk factor for cardiovascular disease and is frequently observed in patients with NAFLD. However, NAFLD may independently contribute to the development of cardiovascular diseases. Despite the expectation of future treatments for NAFLD, current studies are limited by their failure to meet the necessary histological endpoints or by their minimal results.

Upon reviewing the available literature, it becomes evident that MAFLD has been incorporated into various areas of research. However, thus far, there is a lack of comprehensive summaries that integrate the diverse aspects of MAFLD. Therefore, the objective of this literature review is to consolidate recent knowledge and discoveries concerning MAFLD in a single comprehensive resource.

Specifically, this literature review aims to analyze the diagnostic criteria for MAFLD while differentiating it from NAFLD and AFLD. Additionally, it will explore the epidemiology, clinical manifestations, pathogenesis, and management approaches associated with MAFLD.

The literature searches for this review article were made between November 2022 and January 2023. The search words “Metabolic associated fatty liver disease AND (MAFLD AND (Non-alcoholic fatty liver disease OR Non-alcoholic steatohepatitis OR NAFLD OR NASH))” were used in PubMed.

## Definitions

Non-alcoholic fatty liver disease (NAFLD) is characterized by the accumulation of more than 5% hepatic steatosis, excluding other liver diseases and the absence of excessive alcohol consumption (defined as 20 grams per day for women and 30 grams per day for men). Additionally, secondary causes of steatosis, such as viral hepatitis or autoimmune hepatitis, must be ruled out (4, 5).

In contrast, metabolic associated fatty liver disease (MAFLD) encompasses the presence of more than 5% hepatic steatosis without excluding other liver diseases, including alcoholic liver disease. Moreover, the diagnosis of MAFLD requires the presence of a metabolic comorbidity, such as type 2 diabetes mellitus (T2DM), overweight or obesity, or components of the metabolic syndrome (1).

Alcohol-related fatty liver disease (ALD) primarily arises from the consumption of alcohol, resulting in hepatic steatosis (1) (Table 1).

**Table 1:** Summary of definitions.

	NAFLD	MAFLD	ALD
Definition	Presence steatosis with exclusion of other liver disease and excessive alcohol consumption	Presence of steatosis without exclusion of other liver diseases (including alcohol consumption), definition requires presence of metabolic comorbidities (e.g., T2DM).	Steatosis caused by increased alcohol consumption (20 g/d for women and 30 g/d for men).
Subclassifications	NAFL, NASH	N/A	Alcoholic hepatitis
			Alcohol-associated steatosis
			Alcohol-associated steatohepatitis
			Alcohol-associated cirrhosis
Histologic findings	>5% steatosis	>5% steatosis	-
Exclusions	Excessive alcohol consumption	-	-
	Viral hepatitis		
	Autoimmune hepatitis		

### Epidemiology

The incidence of metabolic associated fatty liver disease (MAFLD) remains unknown as it is a recently introduced terminology, lacking current studies reporting its global or regional occurrence. Conversely, a recent meta-analysis and systematic review conducted in September 2022 estimated the incidence of non-alcoholic fatty liver disease (NAFLD) to be 46.9 cases per 1,000 person-years (12).

Over the past two decades, the prevalence of MAFLD has shown an increasing trend. Serial national surveys conducted in the United States indicated a rise in MAFLD prevalence from 28.4% during the period of 1999–2002 to 35.8% during the period of 2011–2016 (13). A meta-analysis published in December 2021 reported the highest prevalence of MAFLD in Europe, followed by Asia and North America. Moreover, 81.59% of individuals diagnosed with NAFLD met the criteria for MAFLD diagnosis based on the current MAFLD definition (95% CI, 66.51%–90.82%). Notably, patients had higher odds of being diagnosed with MAFLD compared to NAFLD (odds ratio, 1.37; 95% CI, 1.16–1.63; *p* < 0.001).

In the United States, the prevalence of MAFLD ranged from 25.9 to 39.1% (14–16). South America is known to have a high prevalence of NAFLD (30.5%); however, there are no studies reporting the prevalence of MAFLD in this continent (17). As for Europe, there is limited research on the epidemiology of MAFLD. A study investigating a large cohort in the United Kingdom (423,252 individuals) found that 38.0% of the participants had MAFLD (18). An extensive systematic review and meta-analysis involving over 13 million individuals in Asia reported a prevalence of 29.62% for MAFLD. A meta-analysis indicated a prevalence of 13.5% for NAFLD in Africa, ranging from 9% in Nigeria to 20% in Sudan (19). The prevalence of MAFLD in the Middle East and North Africa (MENA) region has not yet been documented in studies. Nevertheless, the high prevalence of NAFLD in this region, reported as 31.8%, suggests a substantial burden of MAFLD as well (20).

Overall, the global distribution of MAFLD is uneven, with Europe, Asia, and North America displaying higher prevalence rates compared to other regions. Nevertheless, more research is needed to further investigate the epidemiology and prevalence of MAFLD, particularly in understudied areas such as Africa and the MENA region.

### Pathogenesis

In 1998, a “two-hit” theory was proposed as a pathogenic explanation for non-alcoholic fatty liver disease (NAFLD). According to this theory, the “first hit” involves the accumulation of liver fat, while the “second hit” occurs due to increased levels of inflammatory cytokines, adipokines, mitochondrial dysfunction, and oxidative stress. These factors contribute to the progression from NAFLD to non-alcoholic steatohepatitis (NASH) and advanced fibrosis (21). However, the NAFLD-NASH-HCC sequence exhibits high heterogeneity, making it challenging to incorporate the various molecular and metabolic aspects. NAFLD represents a broad term that encompasses various underlying subtypes (1). Therefore, a “multi-hit” hypothesis has been proposed, which considers multiple processes such as insulin resistance, lipotoxicity, inflammation, cytokine imbalances, activation of innate immunity, and alterations in gut microbiota. This hypothesis takes into account both environmental and genetic factors and aids in understanding the pathogenesis of metabolic associated fatty liver disease (MAFLD) (22). The concept of MAFLD integrates metabolic dysfunction, including hyperglycemia, hypertension, abdominal obesity, and dyslipidemia.

Although the article presents a new hypothesis supporting the use of MAFLD instead of NAFLD, the primary and most significant impact is attributed to hepatic fat accumulation. Epidemiological evidence suggests that individuals with type 2 diabetes mellitus (T2DM) have a higher propensity to develop NAFLD compared to those without this condition. Despite the relatively lower prevalence of type 2 diabetes mellitus (T2DM), there is still evidence supporting the involvement of insulin resistance (IR) in the pathogenesis of NAFLD (23). IR, which is commonly observed in obese and diabetic patients, disrupts hepatic glucose production while promoting lipogenesis (24).

### Glucose metabolism in MAFLD

**Figure 1:**
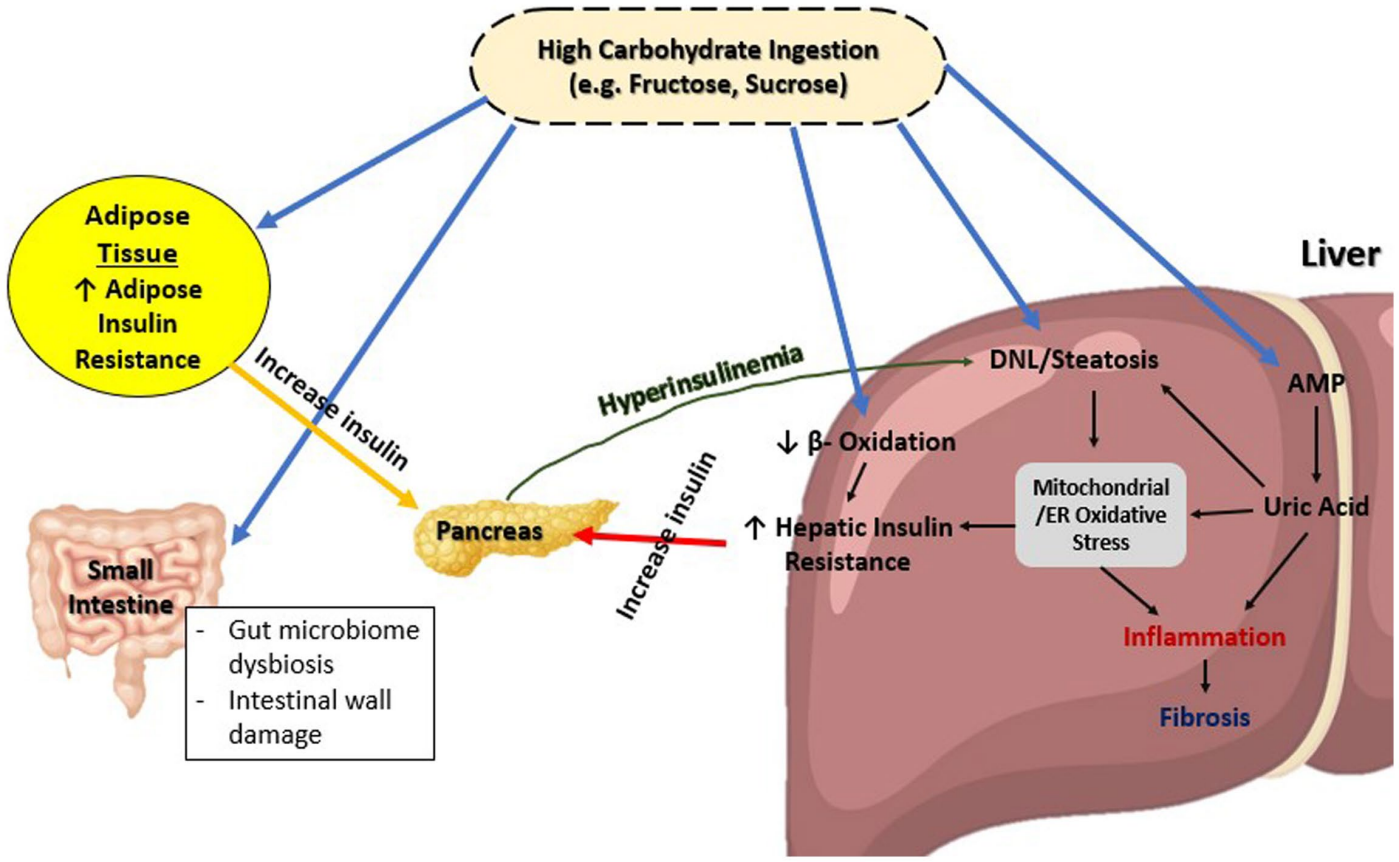
Overview of glucose metabolism in MAFLD. ER, Endoplasmic reticulum; DNL, *De novo* lipogenesis; AMP, Adenosine monophosphate.

As shown on Figure 1, diets high in carbohydrates, such as fructose or sucrose, are strongly associated with NAFLD (25, 26). This increase in blood sugar eventually leads to increased endoplasmic reticulum (ER) and mitochondrial stress, which is known as glucotoxicity. There is evidence that liver cells exposed to high levels of glucose, fructose, or sucrose induce insulin resistance in rodents (27, 28), Consequently, insulin receptor signaling could be suppressed by decreased insulin receptor expression or increased phosphorylation of insulin receptor substrate 1 (IRS-1) (29, 30). In the event that such a condition persists chronically, as in T2DM, it is linked to oxidative stress, inflammation, and further stresses the ER to as depicted in Figure 1 (31).

This chronic low-grade inflammation caused by glucotoxicity and contributing to IR is also shared between NAFLD and T2DM. The process occurs through two classical pathways: nuclear factor Kappa beta (NFκβ) pathway and c-Jun NH2 terminal kinase (JNK) signaling pathway (32, 33). Both these pathways are dependent on the presence of inflammatory regulators such as interleukin-1β (IL-1β), IL-6, tumor necrosis factor (TNF)-alpha, and C-reactive protein (CRP) all of which present in higher level in serum of T2DM patients compared to those without T2DM (34). Moreover, this leads to *de novo* lipogenesis through a variation of pathways, as well as upregulation of lipogenic enzymes genes, which has a downstream effect on insulin sensitivity (35–38).

The presence of chronic hyperglycemia, commonly observed in individuals with type 2 diabetes mellitus (T2DM), is associated with various pathological processes including chronic low-grade inflammation, oxidative stress, endoplasmic reticulum (ER) stress, steatosis, and apoptosis. These interconnected mechanisms provide insight into the pathogenesis of metabolic associated fatty liver disease (MAFLD) as defined by the new criteria. However, further investigation is warranted to elucidate the specific pathways involved in MAFLD, particularly within the context of the updated definition, as previous studies have primarily focused on the older definition of non-alcoholic fatty liver disease (NAFLD). The adoption of the new MAFLD definition has the potential to enhance our understanding of the underlying mechanisms and may offer therapeutic strategies aimed at inhibiting pathological pathways and promoting beneficial pathways in the management of the disease. Continued research in this area is imperative to advance our knowledge and improve clinical outcomes in individuals with MAFLD.

### Fatty metabolism in MAFLD

**Figure 2:**
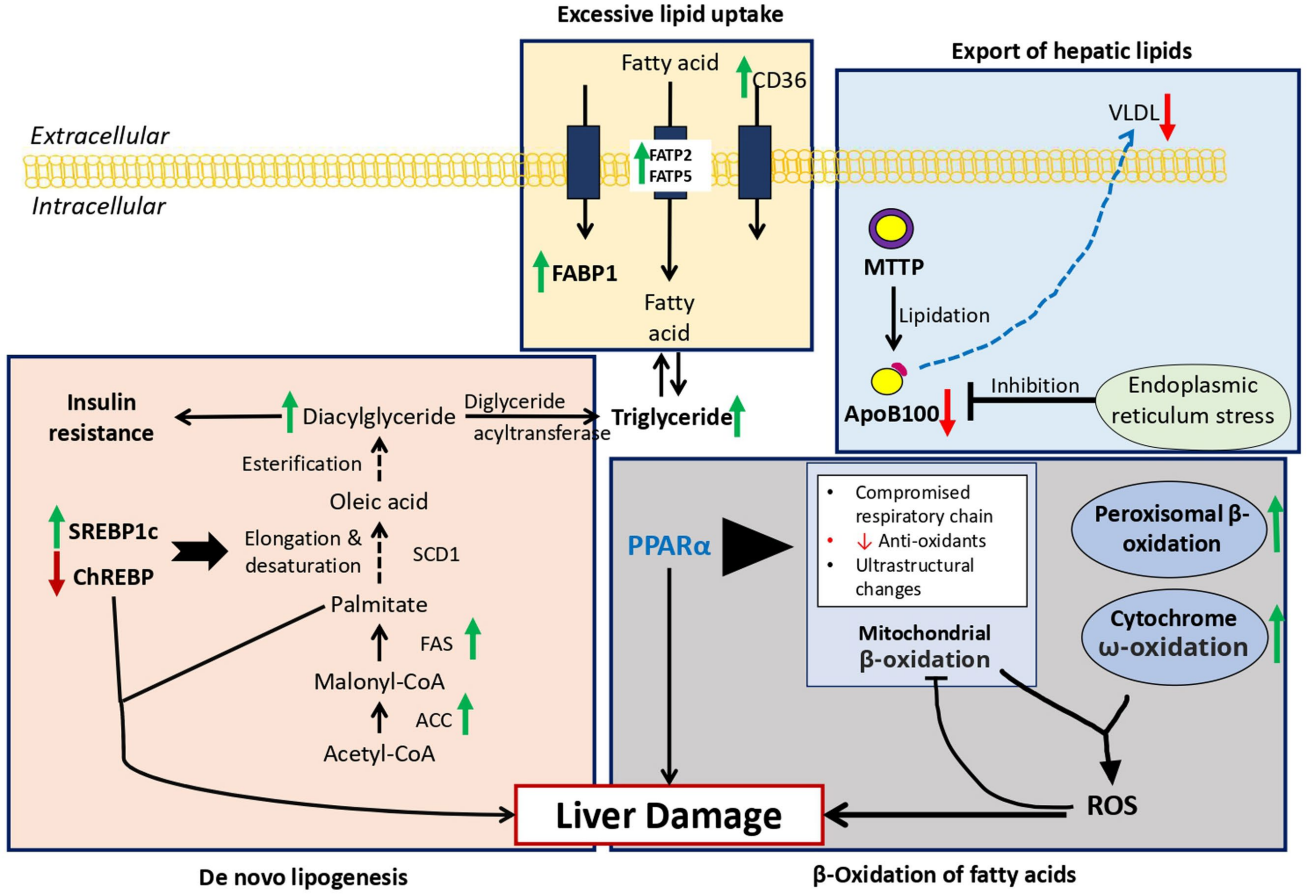
Overview of fat metabolism in MAFLD: Green arrow = Increase expression in MAFLD, Red arrow = Decrease expression in MAFLD. 1. Excessive lipid uptake: Due to increase expression of FABP1, FATP2, FATP5, and CD36 resulting hepatic steatosis. 2. *De novo* lipogenesis: Overexpression of SREBP1c and down regulation of ChREBP result in upregulation of key enzymes in *de novo* lipogenesis resulting in hepatic lipid accumulation. 3. β-Oxidation of fatty acids: Reactive oxygen species produced due to increased ω-oxidation due to fatty acid oxidation inhibits the protective effects of PPARα leading to marked steatosis and inflammation. 4. Export of hepatic lipids: Due to excessive lipid intake, endoplasmic reticulum stress develops leading to decrease in ApoB100 and increase in steatosis. FABP1, Fatty acid binding protein 1; FATP2/5, fatty acid transport proteins 2/5; CD36, cluster of differentiation 36; ACC, acetyl-CoA carboxylase; FAS, fatty acid synthase; SCD1, stearoyl-CoA desaturase-1; SREBP1c, sterol regulatory element-binding protein 1c; ChREBP, carbohydrate regulatory element-binding protein; MTTP, microsomal triglyceride transfer protein, ApoB100.

Hepatic steatosis has been shown to arise within days of high-fat diet in both rodents and humans (39, 40). Shown in Figure 2 are the four main mechanisms causing lipid accumulation in MAFLD: excessive lipid uptake, *de novo* lipogenesis, β-oxidation of fatty acids, and export of hepatic lipids.

#### Excessive lipid uptake

Fatty acid binding protein 1 (FABP1), exclusively expressive in the liver. FABP1 transport fatty acids between organelles and also bind cytotoxic free fatty acid and facilitate their oxidation or incorporation into triglycerides (41). Mice with deleted FABP1 gene had attenuated fasting-induced increase in hepatic triglyceride uptake and oxidation (42). This pathway requires more studies in regard to the effect of antagonism of FABP1 in humans and whether it might yield any therapeutic potential.

Secondly, hepatic lipid uptake is controlled via action of fatty acid transport proteins (FATPs) and cluster of differentiation 36 (CD36), with FATP isoforms 2 and 5 being major isoforms present in the liver (41). Increased expression of FATP5 in humans correlated with higher hepatic steatosis in male MAFLD patients (43). In addition, the level of CD36 protein is increased in the liver in response to high-fat diet feeding (44). According to this research, FATP5, CD36, and hepatic lipotoxicity are related. Although the precise mechanisms used by these proteins have not yet been determined, they provide a potential therapeutic target that can be explored in the future. It is unclear whether these proteins contribute to the progression of MAFLD or whether they have any impact on the disease.

#### *De novo* lipogenesis

There are three enzymes, acetyl-CoA carboxylase (ACC), fatty acid synthase (FAS), and stearoyl-CoA desaturase-1 (SCD1), which regulate *de novo* lipogenesis in the liver. These enzymes result in formation of palmitate, oleate and palmitoleate (45). These fatty acids eventually are either stored as triglycerides or exported as VLDL particles (46). The level of these two molecules leads to hepatic steatosis and hypertriglyceridemia (45). The two transcription factors that regulate the enzymes mentioned before (FAS and SCD1), are sterol regulatory element-binding protein 1c (SREBP1c) and carbohydrate regulatory element-binding protein (ChREBP). Overexpression of SREBP-1c has been linked to upregulation of key enzymes in *de novo* lipogenesis resulting in hepatic lipid accumulation (47). While ChREBP has been linked to normal lipogenic response following the ingestion of carbohydrates such as sucrose and fructose (48). Liver-specific inhibition of ChREBP improves hepatic steatosis and insulin resistance in ob/ob mice (49). Enhanced *de novo* lipogenesis may be responsible for the lipid accumulation in MAFLD and the accumulation of toxic lipid species such as ceramides, which contribute to the transition to NASH. It is imperative that further research is conducted on this pathway as a potential therapeutic target for the treatment of MAFLD.

#### β-oxidation of fatty acids

Normally fatty acid oxidation occurs in mitochondria, while oxidation of very long-chain fatty acids occurs in peroxisomes and then processed by mitochondria (45). During lipid accumulation during high-fat diet, there is an overload of lipid, in which the ω-oxidation by cytochrome P450 enzymes contribute to the fatty acid oxidation. However, the use of this pathways generates large amounts of reactive oxygen species (ROS), leading to inflammation and accelerated progression to NASH (50). Peroxisome proliferator-activated receptor-α (PPARα) is one of the main regulators of fatty acids oxidation in the mitochondria, peroxisomes and cytochromes (51, 52). In mice with knockout hepatic-specific PPARα, there was marked steatosis, inflammation compared to wild-type mice in response to obesity induced by diet, indicating the activation of the ω-oxidation pathway (53). Targeting the PPARα is a therapeutic approach worth considering.

#### Export of hepatic lipids

Another important pathway to be evaluated is the export of hepatic lipids. The main molecules in this process are apolipoprotein B100 (apoB100) and microsomal triglyceride transfer protein (MTTP). In the ER very low-density lipoprotein (VLDL) particles are formed, while MTTP catalyzes the lipidation of apoB100 (51). This VLDL are later secreted into the bloodstream with the help of ApoB100-mediated mechanisms. However, in the case of increased fat intake, the high level of fatty acid promotes ER stress inhibiting apoB100 secretion and causing steatosis (54). NASH patients have lower level of apoB100 synthesis rates which leads to advanced steatosis in such patients as lipid could not be transported (55). The steatosis and lipotoxicity that result from this pathway promote the progression of MAFLD.

### Microbial metabolome and metagenome of MAFLD

Gut microbiota disruption is associated with obesity, type 2 diabetes, fatty liver disease, inflammatory bowel diseases (IBDs), and several forms of cancer. These microbial imbalances may affect multiple biological pathways, including immune function, energy regulation, as well as lipid and glucose metabolism (56). Dysbiosis of the gut plays a key role in the progression and development of MAFLD. This term describes an imbalance in the composition of the gut microbiota and its functions. This imbalance can lead to the production of pro-inflammatory microbial metabolites, such as lipopolysaccharides (LPS), which can trigger inflammation in the liver (57). Dysbiosis can also disrupt the metabolism of bile acids, leading to increased hepatic fat accumulation and inflammation (57). Furthermore, alterations in the gut microbiota can affect the production of short-chain fatty acids (SCFAs), which have a diverse range of metabolic effects (56). Understanding and addressing gut dysbiosis is critical in the context of MAFLD, as it offers potential therapeutic strategies to modulate the gut microbiota and its metabolites, ultimately improving the management of this condition (58).

So far, multiple studies have looked at fecal metagenomic signatures to investigate the genetic pathways that are getting activated in order to gain a better understanding of the etiology of hepatic steatosis (59, 60). In a recent review of the metagenomic, it was found that the production of acetate and ethanol were elevated in normal weight, overweight and obese individuals. Additionally, those who were morbidly obese had elevated carbohydrate, lipids and amino acids metabolism, with increased biosynthesis of branched chain amino acids, aromatic amino acids, lipopolysaccharides and peptidoglycan (61). The possibility of using a diagnostic biomarker instead of invasive methods is thus suggested. However, many of the findings could be explained by the obesity status of the patient, their ethnicity or severity of disease (61). The dietary intake of a subject has been largely absent from many of these studies, which should be considered for future metabolomics and metagenomics (61, 62).

## Genetics and epigenetics

Variations in certain genes may play a role in the pathogenesis of MAFLD. The use of genome-wide and exome-wide association studies (GWAS and EWAS) has uncovered single nucleotide polymorphisms related to MAFLD. These genes are discussed individually in the section below Table 2 provides a summary for the genes and their effects (63).

**Table 2:** Summary of different Genes their SNP and Effect on MAFLD.

Gene	SNP	Effect on MAFLD
PNPLA3	rs738409 C > G	Increases risk of MAFLD by promoting liver fat accumulation
TM6SF2	rs58542926 C > T	Increases risk of MAFLD by impairing VLDL and steatosis
MBOA7	RS651738 C > T	Increases risk of MAFLD by promoting steatosis and fibrosis
MARC1	RS2642438 A > G	Appears to be protective against MAFLD by reducing fibrosis and fat accumulation
HSD17B13	rs72613567 T > TA and rs62305723	Associated with less hepatic inflammation and cellular ballooning
FNDC5	NA	Potential therapeutic opportunity for MAFLD
IFNL3/4	rs12979860 and rs368234815	Linked with higher degrees of liver inflammation and fibrosis
FGF21	rs838133	Associated with more hepatic inflammation in MAFLD individuals
CNV	NA	Believed to influence MAFLD pathogenesis, but more research is needed
Epigenetics	NA	DNA methylation is the most significant factor in MAFLD pathogenesis

### PNPLA3

Patatin-like phospholipase domain-containing 3 (PNPLA3) encodes a protein that regulates lipolysis and lipogenesis in fat and liver cells. The SNP rs738409 C > G of this gene has been identified as the most robust genetic variant associated with MAFLD. The mechanism by which this variant promotes MAFLD pathogenesis is believed to be due to sequestration of adipose triglyceride lipase co-activator (CGI-58) leading to liver fat accumulation. This variant increased MAFLD risk but not that of fatty liver disease without metabolic dysfunction. Thus, metabolic dysfunction maybe a prerequisite for this gene variant to contribute to fatty liver disease (63).

### TM6SF2

Transmembrane 6 superfamily member 2 (TM6SF2) encodes a protein found in the endoplasmic reticulum membrane. The variant rs58542926 C > T is linked with hepatic steatosis, impaired VLDL, and thus increases the risk of MAFLD. However, the association of this variant with fatty liver disease, just like that of PNPLA3, was seen only in subjects with metabolic dysfunction (63).

### MBOAT7

Membrane-bound O-acyltransferase domain-containing 7 (MBOAT7) also encodes a protein in the endoplasmic reticulum membrane. The rs651738 C > T variant of this gene increases the risk of MAFLD. This effect is mediated by steatosis and fibrosis. This is evident in a study which found out that carriers of this variant had more liver fat, increased liver injury, and higher risk of fibrosis compared to non-carriers (63).

### MARC1

Mitochondrial amidoxime reducing component 1 (MARC1) encodes a protein found in the outer mitochondrial membrane. The rs2642438 A > G variant of this gene seems to be protective from MAFLD. Recent studies found out that subjects with this variant had less degree of fat accumulation in the liver, liver fibrosis, and cirrhosis. However, further studies are needed to understand how exactly MARC1 influences the pathogenesis of MAFLD (63).

### HSD17B13

Hydroxysteroid 17-β dehydrogenase 13 (HSD17B13) is a liver-specific lipid droplet-associated protein. The 2 variants of this gene rs72613567 T > TA and rs62305723 are associated with less hepatic inflammation and cellular ballooning. However, mice studies consequently showed contradicting results with no protective effects. Therefore, more research needs to be done to study the effect of this gene on MAFLD (63).

### FNDC5

Fibronectin type III domain-containing 5 (FNDC5) encodes a protein that is cleaved in muscle cells, and forms irisin, which is secreted into the blood. Irisin has a negative association with triglyceride content in liver steatosis and hepatic steatosis. Administration of nicotinamide riboside (NR) had led to an increase in plasma irisin level leading to better insulin sensitivity, less hepatic steatosis, steatohepatitis, and fibrosis. Therefore, this gene may be a promising therapeutic potential for MAFLD (63).

### IFNL4

The interferon lambda type 3 and 4 (IFNL3/4) are cytokines induced by viral infection and are associated with immune and inflammatory response. According to studies, IFNL4 rs12979860 and rs368234815 variants were linked with higher degree of liver inflammation and fibrosis in MAFLD subjects from Europe (63).

### FGF21

Fibroblast growth factor 21 (FGF21) is a protein secreted from liver cells that regulates metabolism, Nevertheless, it is still not known whether the impact of this protein on metabolism is beneficial or not. Recently, it was discovered that the rs838133 variant of this gene was associated with more hepatic inflammation in MAFLD individuals (63).

### CNV

Copy number variation (CNV) plays an important role in MAFLD. CNV is a form of genetic alterations that differs from SNPs in terms of size and rates of mutation. Recent research suggests that CNV influences gene expression and contributes to a variety of diseases, including MAFLD. However, the evidence that CNV is correlated with MAFLD is limited. A study in 2014 demonstrated that four rare and novel CNVs were linked with MAFLD. It has also been shown that XOP4 gene CNVs are associated with hepatic fibrosis, although more understanding is necessary to understand how CNVs influence MAFLD pathogenesis (63).

Several epigenetic factors contribute to the pathogenesis of MAFLD, including DNA and RNA methylation, histone modification, noncoding RNA, microRNA, and circular RNA. Of these, methylation of DNA is the most significant (63).

## MAFLD diagnosis

In contrast to non-alcoholic fatty liver disease (NAFLD), which is primarily diagnosed by excluding excessive alcohol intake and viral liver disease, metabolic associated fatty liver disease (MAFLD) follows a more inclusive approach where exclusion of other hepatic conditions is not a prerequisite for diagnosis. The diagnostic criteria for MAFLD include the presence of hepatic steatosis confirmed by biopsy, imaging, or blood markers, along with the coexistence of one or more conditions such as overweight/obesity, type 2 diabetes mellitus, or evidence of metabolic dysregulation in lean individuals.

The American Association of Clinical Endocrinology (AACE) and European Association for the Study of the Liver (EASL) recently released guidelines outlining various imaging techniques for diagnosing hepatic steatosis. These include liver ultrasound (US), Fibroscan, computed tomography (CT) scan, and magnetic resonance imaging (MRI). However, it should be noted that liver biopsy remains the gold standard for accurately diagnosing liver steatosis (64). Liver ultrasound, although commonly used, exhibits limitations in terms of sensitivity, particularly in detecting mild-to-moderate steatosis. Additionally, liver ultrasound is operator-dependent and does not provide information regarding the severity of liver fibrosis unless cirrhosis is present (64). MRI, on the other hand, is an expensive option and is typically reserved for selected individuals under the guidance of hepatologists (64).

There are many serum biomarkers that have valuable diagnostic and prognostic value for steatosis. These include total cholesterol, triglycerides, insulin resistance, C-peptide, γ-glutamyl transferase (GGT), ALT, and IL-6 (65, 66). Some of these markers have been implicated in non-invasive methods to diagnose liver fibrosis. Among the non-invasive methods available, the Fibrosis-4 index (FIB-4) has demonstrated validation in diagnosing clinically significant fibrosis (67). This index incorporates age, serum aminotransferase levels (AST and ALT), and platelet count in its calculation (age in years × AST in U/L)/(platelet count in 10^9^/L × ALT^0.5^ in U/L). FIB-4 has prognostic capabilities in predicting changes in liver fibrosis over time and enables risk stratification for future liver-related morbidity and mortality (61). This index incorporates age, serum aminotransferase levels (AST and ALT), and platelet count in its calculation (age in years × AST in U/L)/(platelet count in 10^9^/L × ALT^0.5^ in U/L). FIB-4 has prognostic capabilities in predicting changes in liver fibrosis over time and enables risk stratification for future liver-related morbidity and mortality (64). However, it is important to note that FIB-4 and similar blood-based measures have limitations in terms of sensitivity and positive predictive value (PPV), making them less reliable for identifying advanced fibrosis. Nevertheless, these scores exhibit good specificity and negative predictive value (NPV), which are valuable for ruling out advanced liver fibrosis. Further research is warranted to develop more accurate diagnostic tools for advanced fibrosis in MAFLD.

The initial step in assessing individuals with a high risk of metabolic associated fatty liver disease (MAFLD) involves evaluating their risk of liver fibrosis using non-invasive techniques such as liver ultrasound (US) and Fibroscan, also known as transient elastography (TE). Among these techniques, Fibroscan has demonstrated superiority over US in clinical diagnosis due to its ability to quantitatively assess both liver fat and fibrosis (67).

For individuals with a high pre-test probability of MAFLD, as indicated in the algorithm Figure 2, the Fibrosis-4 index (FIB-4) can be utilized to stratify their fibrosis risk without the necessity of diagnosing hepatic steatosis through liver US. The fibrosis risk is categorized into three groups: low risk (FIB-4 < 1.3), high risk (FIB-4 > 2.67), and indeterminate risk (FIB-4 1.3–2.67). In the indeterminate and high-risk groups (FIB-4 > 2.67), a second-level test, such as Fibroscan for liver stiffness measurement (LSM) or the enhanced liver fibrosis blood (ELF) test, should be conducted. Based on the LSM or ELF values obtained, individuals classified as indeterminate risk (LSM 8–12 kPa or ELF 7.7–9.8) or high risk (LSM > 12 kPa or ELF >9.8) for liver fibrosis should be referred to hepatologists for further assessment and potential consideration of liver biopsy, which remains the gold standard for diagnosing non-alcoholic steatohepatitis (NASH) (64). However, it is important to note that liver biopsy is invasive, subject to interpretation errors, and impractical for large target populations; therefore, it should not be employed as a screening tool for the diagnosis of MAFLD (64).

There are various indices used to estimate the likelihood of liver steatosis. One well known index is the Fatty Liver Index (FLI). This is a simple and accurate algorithm that takes into consideration body mass index (BMI), waist circumference, triglycerides, and gamma-glutamyl-transferase (GGT). The score ranges from 0 to 100. According to Bedogni et al., an FLI score < 30 with a negative likelihood ratio of 0.2 rules out liver steatosis. On the other hand, an FLI score ≥ 60 with a positive likelihood ratio of 4.2 rules in fatty liver (68).

Another validated screening tool for MAFLD is the Hepatic Steatosis Index (HSI). This algorithm takes into consideration Aspartate Aminotransferase (AST)/Alanine Aminotransferase (ALT) ratio, BMI, sex, and the presence of diabetes mellitus, as illustrated; (HSI) = 8 × (ALT/AST ratio) + BMI (+2, if female; +2, if diabetes mellitus). Values < 30 ruled out MALFD with a sensitivity of 93.1%, whereas values >36 ruled in MAFLD with a specificity of 92.4% (69).

Controlled Attenuation Parameter (CAP) is an ultrasound-based non-invasive technique that measures attenuation levels in the liver. This method has a sensitivity of 87% and a specificity of 91% for detecting mild hepatic steatosis (70).

Computed Tomography (CT) scan is also used for the evaluation of MAFLD. This is based on measuring the attenuation value of liver parenchyma, expressed as Hounsfield units (HU). The most commonly used CT scan-based technique for assessing liver steatosis is measuring the attenuation difference between liver and spleen (CT_L-S_) on unenhanced CT. In normal circumstances, attenuation value of liver parenchyma is slightly higher than that of the spleen. As hepatic steatosis progresses in the liver, the liver’s attenuation value decreases, thus decreasing the CT_L-S_ value. This method is regarded as a fairly accurate tool with sensitivity and specificity values of 82 and 100%, respectively for moderate-to-severe liver steatosis (71).

## Associations with other conditions

The presence of metabolic-associated fatty liver disease (MAFLD) is commonly linked to a heightened susceptibility to mortality across various causes [hazard ratio (HR) = 1.24; 95% confidence interval (CI) = 1.13–1.34] (72). Below are several conditions that can lead to mortality and their associations with MAFLD.

### CKD

In comparison to non-alcoholic fatty liver disease (NAFLD), the newly proposed metabolic dysfunction-associated fatty liver disease (MAFLD) exhibits enhanced diagnostic accuracy for identifying patients with chronic kidney disease (CKD). A comprehensive study involving a total of 12,571 patients was conducted to investigate this phenomenon. The study revealed that individuals diagnosed with MAFLD exhibited a significantly lower estimated glomerular filtration rate (eGFR) value (74.96) when compared to those diagnosed with NAFLD (74.46; *p* < 0.001). Additionally, the prevalence of CKD was found to be higher among patients diagnosed with MAFLD (29.6%) compared to those diagnosed with NAFLD (25.6%; *p* < 0.05). Further analysis indicated that MAFLD patients exhibiting severe fibrosis (defined as the product of the NAFLD fibrosis score and 0.676) were 1.34 times more likely to develop CKD in comparison to patients with non-severe MAFLD. Remarkably, this association held true regardless of the patients’ age, gender, ethnicity, alcohol consumption, or diabetes status (73). In a cohort study involving 27,371 participants, a significant association was observed between MAFLD and the incidence [hazard ratio (HR) = 1.24, 95% confidence interval (CI) = 1.14–1.36, *p* = 0.001] as well as the prevalence [odds ratio (OR) = 1.83, 95% CI = 1.66–2.01, *p* = 0.001] of CKD (74). In a large-scale nationwide cohort study encompassing over 250,000 participants and employing a median follow-up period of 5.1 years, it was observed that individuals diagnosed with MAFLD displayed a notably elevated risk of developing incident CKD when compared to those diagnosed with NAFLD. The HR for incident CKD in the MAFLD group was found to be 1.18 (95% confidence interval: 1.01–1.39, *p* = 0.04), indicating a statistically significant association between MAFLD and CKD occurrence (75). Moreover, a comprehensive meta-analysis and systematic review was conducted to assess the association between MAFLD and CKD in comparison to individuals without MAFLD. The analysis revealed that MAFLD was significantly associated with an increased risk of developing CKD (HR: 1.53, 95% CI: 1.38–1.68). This finding underscores the substantial impact of MAFLD on CKD susceptibility and highlights the importance of recognizing and managing MAFLD as a potential risk factor for CKD development (72). Another longitudinal study spanning a 10-year period, a cohort of 28,890 Japanese patients was examined to investigate the association between MAFLD and the development of CKD. After adjusting for potential confounding factors such as age, gender, eGFR, smoking, and comorbidities, it was revealed that MAFLD served as an independent predictor for the incidence of CKD. The HR for CKD development associated with MAFLD was estimated to be 1.12 (95% confidence interval: 1.02–1.26, *p* = 0.027), indicating a statistically significant relationship between MAFLD and the risk of CKD, even when accounting for the aforementioned variables (76).

### Liver disease

A study conducted in Japan investigated the diagnostic capabilities MAFLD in comparison to NAFLD for detecting significant liver stiffness. The study revealed that MAFLD exhibited a higher sensitivity (93.9%) in identifying significant liver stiffness compared to NAFLD (73.0%). In a meta-analysis and systematic review, the results indicated that patients with MAFLD alone had nearly four times the risk of liver fibrosis in comparison to those with NAFLD alone [relative risk (RR), 4.2; 95% confidence interval (CI), 1.3–12.9] (77). The results of another study, a meta-analysis and systematic review, indicated that patients with MAFLD alone had nearly four times the risk of liver fibrosis as those with NAFLD alone (RR, 4.2, 95% CI, 1.3–12.9) (78). Furthermore, an extensive study conducted using the UK Biobank dataset found that individuals diagnosed with MAFLD were at a significantly higher risk of developing cirrhosis [hazard ratio (HR), 2.77; 95% CI, 2.29–3.36] and liver cancer (HR, 1.59) compared to those without the condition. This study emphasized the increased susceptibility of MAFLD patients to the development of severe liver complications (18, 79).

### Cardiovascular disease

Metabolic dysfunction-associated fatty liver disease (MAFLD) is recognized as a prominent contributor to cardiovascular disease (CVD)-related mortality (80). A nationwide cohort study was conducted involving a cohort of over 8.9 million individuals from Korea, with a median follow-up duration of 10.1 years, to investigate the risk of developing CVD events such as myocardial infarction, ischemic stroke, heart failure, or CVD-related mortality. After adjusting for age, gender, and other relevant variables, participants diagnosed with MAFLD alone exhibited a significantly higher risk of developing CVD events compared to those with non-alcoholic fatty liver disease (NAFLD) alone [hazard ratio (HR) 1.43 vs. 1.09]. Notably, the study indicated that the risk of cardiovascular events varied among different subtypes of MAFLD. The overweight-MAFLD group displayed a risk of 1.16 [95% confidence interval (CI) 1.15–1.18], the lean-MAFLD group exhibited a risk of 1.23 (95% CI 1.20–1.27), and the diabetes-MAFLD group had a risk of 1.82 (95% CI 1.80–1.85), irrespective of comorbidities. These findings suggest that individuals with diabetic MAFLD are more susceptible to developing cardiovascular events compared to those with overweight/obese or lean MAFLD (81). A meta-analysis and systematic review reported that MAFLD significantly increases the risk of cardiovascular disease (HR 1.49, 95% CI 1.34–1.64, *p* < 0.01), CVD-related mortality (HR 1.28, 95% CI 1.03–1.53, *p* = 0.04), and stroke (HR 1.55, 95% CI 1.37–1.73, *p* < 0.01) when compared to non-MAFLD individuals (72). Moreover, another meta-analysis and systematic review concluded that patients with MAFLD alone face a 3.41 times greater risk of cardiovascular mortality compared to those with NAFLD alone [relative risk (RR) 3.41, 95% CI 2.31–5.02] (82).

### Cancer

An extensive analysis conducted on a large population of more than 350,000 individuals in the United Kingdom revealed associations between MAFLD and several types of cancer, including gallbladder cancer (HR 2.20, 95% CI 1.14–4.23), kidney cancer (HR 1.77, 95% CI 1.49–2.11), thyroid cancer (HR 1.69, 95% CI 1.20–2.38), esophageal cancer (HR 1.48, 95% CI 1.25–1.76), pancreatic cancer (HR 1.31, 95% CI 1.10–1.56), bladder cancer (HR 1.26, 95% CI 1.11–1.43), colorectal and anal cancers (HR 1.14, 95% CI 1.06–1.23), endometrial cancer (HR 2.36, 95% CI 1.99–2.80), and breast cancer (HR 1.19, 95% CI 1.11–1.27) (18, 79). A retrospective study involving 124 individuals independently linked MAFLD with colorectal adenoma [odds ratio (OR) 3.19, 95% CI 1.49–7.07, *p* = 0.003] (83). Moreover, a large nationwide cohort study indicated that individuals with MAFLD had a higher risk of developing colorectal cancer compared to those without NAFLD or MAFLD (HR 1.32, 95% CI 1.28–1.35), whereas individuals with NAFLD had a slightly lower risk (HR 1.16, 95% CI 1.06–1.28) (81). Evidence from a meta-analysis and systematic review demonstrated that severe MAFLD is independently associated with colorectal cancer when compared to non-severe MAFLD (OR 3.03, 95% CI 2.02–4.53) (84). Individuals with MAFLD also face an increased risk of cancer-related mortality compared to those without MAFLD (HR 1.27, 95% CI 1.01–1.54) (72).

### Diabetes

MAFLD is independently associated with an elevated risk of developing diabetes. A cohort study conducted in China reported that individuals diagnosed with MAFLD had more than double the risk of developing diabetes compared to those without fatty liver (RR 2.08, 95% CI 1.72–2.52) (85).

## Management of MAFLD

To achieve therapeutic efficacy, an optimal treatment approach for metabolic dysfunction-associated fatty liver disease (MAFLD) should target various aspects such as steatosis reduction, mitigation of liver damage, alleviation of metabolic consequences associated with the disease, as well as addressing cardiovascular risk. While numerous compounds are currently under investigation in clinical trials for pharmacological management of MAFLD, none have been specifically approved for treatment (86). To achieve therapeutic efficacy, an optimal treatment approach for metabolic dysfunction-associated fatty liver disease (MAFLD) should target various aspects such as steatosis reduction, mitigation of liver damage, alleviation of metabolic consequences associated with the disease, as well as addressing cardiovascular risk. While numerous compounds are currently under investigation in clinical trials for pharmacological management of MAFLD, none have been specifically approved for treatment (80).

Since cardiovascular events represent a leading cause of mortality among MAFLD patients, the implementation of cardioprotective medications may potentially enhance survival rates (80).

Furthermore, as metabolic syndrome is included as one of the diagnostic criteria for MAFLD, dietary modifications may prove beneficial for patients. Meta-analyses suggest that a 7–10% weight reduction resulting from caloric restriction can significantly improve liver steatosis, as indicated by liver enzyme activity, histological assessment of steatosis and inflammation, albeit with less certainty regarding its impact on fibrosis (87). Currently, four medications, namely orlistat, naltrexone extended-release/bupropion extended-release, phentermine/topiramate controlled-release, and liraglutide, are available for weight loss through appetite reduction or fat absorption inhibition (88, 89). A hypocaloric diet, aiming for gradual weight loss (up to 1 kg/week) with a 500–1,000 kcal deficit, is recommended as part of lifestyle management for MAFLD.

Although existing research does not strongly support the effectiveness of a specific dietary strategy, it is worth noting that MAFLD patients often consume high-energy diets low in micronutrients such as fresh fruit, fiber, green vegetables, and omega-3 polyunsaturated fatty acids, while frequently consuming sugar-sweetened beverages and foods high in saturated fat and cholesterol (90). Consequently, the Mediterranean diet, emphasizing increased consumption of fruits, vegetables, whole grains, and olive oil, with reduced carbohydrate intake and increased intake of monounsaturated and omega-3 fatty acids, is frequently recommended (91, 92). Bariatric surgery is another option for weight reduction; however, its invasive nature and potential side effects, including malabsorption leading to severe fibrosis, cirrhosis, and liver failure, necessitate careful consideration (93).

Given the involvement of insulin resistance in the pathophysiology of MAFLD, antidiabetic medications have demonstrated effectiveness in managing the disease (94). Insulin sensitizers such as pioglitazone and metformin are recommended options. Pioglitazone is advised for individuals with confirmed metabolic-associated steatohepatitis, while metformin has shown to significantly improve body composition and liver function in non-diabetic MAFLD patients according to a meta-analysis (94). Promisingly, new anti-diabetic drugs like sodium-glucose cotransporter 2 (SGLT2) inhibitors and glucagon-like peptide 1 receptor agonists (GLP-1RAs) are being investigated for their potential to reverse liver steatosis and halt the progression of severe fibrosis (80, 94). GLP-1RAs, in particular, have demonstrated the ability to reduce significant cardiovascular risks while improving liver histology in MAFLD patients (95). SGLT2 inhibitors, on the other hand, appear to reverse metabolic and hepatic abnormalities associated with MAFLD (96, 97).

Dyslipidemia management in MAFLD often involves the prescription of statins, which are widely used lipid-lowering medications (98). Animal studies have shown that statins can ameliorate hepatic lipotoxicity, inflammatory responses, oxidative stress, and fibrosis associated with metabolic-associated steatohepatitis (99). Additionally, statins offer protection against cardiovascular diseases, making them potentially valuable in reducing associated morbidity and mortality (100).

Vitamin D and E have also been considered as therapeutic options for MAFLD management. Vitamin D exhibits anti-inflammatory, anti-fibrotic, and insulin-sensitizing properties (101). Supplementation with vitamin D has demonstrated beneficial effects on glycemic control and insulin sensitivity in MAFLD patients (102). Population studies have shown an association between vitamin D deficiency and higher prevalence of MAFLD (103). Population studies have shown an association between vitamin D deficiency and higher prevalence of MAFLD (104).

Population studies have shown an association between vitamin D deficiency and higher prevalence of MAFLD (105).

Presently, various pharmaceutical agents targeting different biological pathways are under investigation. For instance, the GLP-1 receptor agonist semaglutide has shown promising results in improving non-alcoholic steatohepatitis (NASH) during phase 2 trials (106). Other weight loss agents, including GLP-1 RA/gastric inhibitory polypeptide (e.g., tirzepatide) analogs and GLP-1 RA/glucagon agonists (e.g., cotadutide), are also undergoing clinical trials to assess their effects on NASH (107, 108). Medications such as pioglitazone, which improve steatohepatitis, fibrosis, and insulin resistance, offer an alternative strategy and are currently being tested in phase 3 trials (109). An example of such a drug is Lanifibranor, a pan-PPAR (peroxisome proliferator–activated receptor) agonist, which affects crucial metabolic, inflammatory, and fibrogenic processes in NASH development (110). Other strategies involve the use of FXR agonists, primarily with antifibrotic effects, and thyroid hormone receptor agonists, which primarily improve steatohepatitis (111). In the interim, the use of diabetes medications like pioglitazone or GLP-1 RAs, with semaglutide having the strongest evidence, should be considered more frequently by clinicians, as these medications have demonstrated the ability to reverse steatohepatitis in controlled clinical trials involving subjects with or without diabetes over a period of 1.5 to 3 years (112, 113). Additionally, vitamin E has shown benefits for individuals with NASH who do not have diabetes (114). However, it is important to note that the U.S. Food and Drug Administration (FDA) has not approved these substances for the management of NASH.

Numerous studies emphasize the crucial role of lifestyle modifications in the treatment of Metabolic Associated Fatty Liver Disease (MAFLD) (115, 116). Recent research underscores the effectiveness of lifestyle changes combined with weight loss, revealing a clear dose–response relationship, where a 9% reduction in body weight significantly improves MAFLD, and a 10% decrease results in a 45% reduction in liver fat (117–119). Randomized controlled trials support the notion that lifestyle adjustments lead to weight loss, increased MAFLD activity score, and reduced hepatic triglyceride levels as measured by MRI scans (120–122). Additionally, a systematic review established a positive association between a sedentary lifestyle and MAFLD prevalence (41).

Physical activity is a non-pharmacological approach that provides benefits in MAFLD treatment. Regular exercise reduces oxidative stress, inflammation, and liver damage markers, including ALT and AST (123). It also promotes a negative energy balance and aids in weight and body fat reduction (121). Aerobic exercise activates AMPK and reduces malonyl CoA in MAFLD, enhancing mitochondrial fatty acid transport and oxidation (124). As a result of elevated lipogenesis, patients with MAFLD accumulate intrahepatic lipids and have high circulating triglyceride levels, which disrupt insulin action and worsen the condition (125). Physical activity, both aerobic and resistance, disrupts this cycle, improving lipid oxidation and glucose control (126).

## Conclusion

In conclusion, the diagnosis of NAFLD lacks inclusion of metabolic diseases in its definition, necessitating the adoption of a new term, MAFLD, that provides a more comprehensive understanding of the disease and its interactions with various physiological processes. This improved understanding facilitates better patient classification, identification of risk factors, disease control, and the development of targeted management plans based on the affected pathways. MAFLD, characterized by hepatic fat accumulation resulting from metabolic dysregulation, is a prevalent global disorder closely associated with insulin resistance, obesity, and type 2 diabetes. Early detection through imaging, liver biopsy, or non-invasive scoring methods is crucial due to the potential progression of MAFLD to severe conditions such as non-alcoholic steatohepatitis, cirrhosis, and liver cancer. Treatment options range from lifestyle modifications to pharmaceutical interventions, with bariatric surgery being a potential option for obese individuals. Recognizing the interplay between MAFLD and other metabolic diseases is essential for improving overall health outcomes and reducing complications. Further investigation is necessary to comprehend the pathogenesis of the disease, develop new therapeutic strategies for its prevention or delayed progression, and identify biomarkers for early diagnosis and prognosis.

## Author contributions

MHab: Investigation, Methodology, Writing – original draft. KJ: Investigation, Methodology, Writing – original draft. AO: Investigation, Methodology, Writing – original draft, Writing – review & editing. MHai: Writing – original draft. MM: Conceptualization, Data curation, Funding acquisition, Investigation, Methodology, Resources, Supervision, Validation, Writing – original draft, Writing – review & editing. A-NE: Conceptualization, Writing – review & editing.
