# Correction: Metabolic-associated fatty liver disease: a selective review of pathogenesis, diagnostic approaches, and therapeutic strategies

**DOI:** 10.3389/fmed.2025.1704750

**Published:** 2025-10-01

**Authors:** Mohammad Habibullah, Khaleed Jemmieh, Amr Ouda, Mohammad Zulqurnain Haider, Mohammed Imad Malki, Abdel-Naser Elzouki

**Affiliations:** ^1^College of Medicine, QU Health, Qatar University, Doha, Qatar; ^2^Internal Medicine Department, Hamad General Hospital, Doha, Qatar; ^3^Weill Cornell Medical Qatar, Doha, Qatar

**Keywords:** metabolic associated fatty liver disease (MAFLD), non-alcoholic fatty liver disease (NAFLD), diagnostic criteria, hepatic steatosis, nonalcoholic steatohepatitis (NASH), epidemiology, clinical manifestations, pathogenesis

An incorrect Funding statement was provided. The correct funder is “Medical Research Center of Hamad Medical Corporation-Qatar.” The correct funding statement should read:

We would like to acknowledge Medical Research Center of Hamad Medical Corporation-Qatar for funding this article.

The original version of this article has been updated.

